# Advanced Microstructural Investigation of the Endodontic Sealing Ability of Three Different Obturation Techniques

**DOI:** 10.3390/dj14010009

**Published:** 2025-12-23

**Authors:** Mihaela Păstrav, Radu Marcel Chisnoiu, Marioara Moldovan, Lucian Barbu Tudoran, Ioan Petean, Andrea Maria Chisnoiu, Ovidiu Păstrav

**Affiliations:** 1 Department of Orthodontics, Faculty of Dental Medicine, “Iuliu Hațieganu” University of Medicine and Pharmacy, 8 Victor Babeș Street, 400012 Cluj-Napoca, Romania; mihaela.pastrav@umfcluj.ro; 2 Department of Odontology, Endodontics and Oral Pathology, Faculty of Dental Medicine, “Iuliu Hațieganu” University of Medicine and Pharmacy, 8 Victor Babeș Street, 400012 Cluj-Napoca, Romania; ovidiu.pastrav@umfcluj.ro; 3 Department of Polymer Composites, Raluca Ripan Institute for Research in Chemistry, Babeș-Bolyai University, 30 Fantanele Street, 400294 Cluj-Napoca, Romania; 4 Faculty of Biology and Geology, Babeș-Bolyai University, 44 Gheorghe Bilaşcu Street, 400015 Cluj-Napoca, Romania; lucian.barbu@ubbcluj.ro; 5 National Institute for Research and Development of Isotopic and Molecular Technologies, 65-103 Donath Street, 400293 Cluj-Napoca, Romania; 6 Faculty of Chemistry and Chemical Engineering, Babeș-Bolyai University, 11 Arany Janos Street, 400028 Cluj-Napoca, Romania; ioan.petean@ubbcluj.ro; 7 Department of Prosthodontics, Faculty of Dental Medicine, “Iuliu Hațieganu” University of Medicine and Pharmacy, 8 Victor Babeș Street, 400012 Cluj-Napoca, Romania; maria.chisnoiu@umfcluj.ro

**Keywords:** endodontic sealing, single cone, lateral compaction, warm vertical condensation, SEM microscopy, sealer thickness

## Abstract

**Objectives:** This study evaluated and compared the sealing ability and elemental composition of a resin-based endodontic sealer (AH Plus) used with three root canal obturation techniques: single cone (SC), lateral compaction (LC), and warm vertical condensation (WVC). The investigation focused on microstructural characteristics, interfacial integrity, and elemental distribution within filled root canals. **Material and Methods:** Sixty extracted single-root teeth were instrumented using the ProTaper Gold system and randomly assigned to three groups (*n* = 20) according to the obturation technique. The AH Plus Jet sealer was applied in all cases. Following obturation, samples were subjected to radiographic investigation and analyzed using optical microscopy and scanning electron microscopy (SEM) coupled with energy-dispersive X-ray spectroscopy (EDX) to assess the sealing performance and chemical composition. **Results:** Radiographic and microscopic assessments indicated that the SC method showed strong gutta-percha adhesion to dentin with a thin cement layer, whereas WVC provided excellent adaptation and penetration of gutta-percha. The LC technique demonstrated good adhesion but displayed occasional structural irregularities. SC has the thicker adhesion layer with uneven distribution regarding coronal, median, and apical, regions ranging from 45 to 80 μm, while WVC ensures a thin and uniform sealing layer of about 35 μm in all regions. SEM and EDX analyses detailed the interfacial microstructure and confirmed the presence of carbon (C), oxygen (O), calcium (Ca), zinc (Zn), barium (Ba), and sulfur (S) across all groups. **Conclusions:** All three obturation techniques (SC, WVC, LC) achieved effective sealing when combined with the AH Plus sealer. The main difference between the methods consists of the sealer layer thickness and its even distribution regarding gutta-percha cones.

## 1. Introduction

Over the past several decades, the field of endodontics has undergone a paradigm shift driven by advances in biomaterial science, analytical imaging, and minimally invasive techniques. A principal focus of contemporary endodontic research has been the development of obturation materials capable of achieving superior biointegration, dimensional stability, and long-term periapical healing. Numerous investigations have sought to refine the physicochemical and biological characteristics of endodontic sealers and core filling materials, with the overarching objective of enhancing the biofunctional interface between the filling material and the dentinal substrate while simultaneously improving prognostic predictability over extended clinical follow-up periods [1,2]. Among the array of biomaterials evaluated, gutta-percha has retained its status as the benchmark obturation material due to its favorable thermoplasticity, chemical inertness, and enduring clinical success. Despite the emergence of novel bioceramic and resin-modified alternatives, gutta-percha remains the most widely adopted core filling medium, serving as the principal component in various obturation techniques designed to create an impervious seal along the root canal system.

Endodontic obturation materials fulfill a dual biological and mechanical function: they must provide an effective apical and coronal barrier to prevent microbial penetration while simultaneously preserving the periapical tissue integrity [3]. Ideally, these biomaterials should be restricted within the endodontic lumen to ensure hermetic sealing [4]. The obturation process must facilitate an impeccable seal to prevent the ingress of percolating fluids that carry nutrients and microbial pathogens into the root canal system. The integrity of this seal is essential, as any microleakage or percolation of fluids containing nutrients and microbial agents can precipitate persistent intraradicular infections and complex endodontic pathologies [5,6]. Consequently, obturation protocols have evolved toward precision-based thermo plasticized techniques in which gutta-percha cones are adapted three-dimensionally within the canal space, their sealing performance augmented through the application of endodontic sealers [7].

Among these, resin-based sealers such as AH Plus—a triethoxysilane-modified epoxy resin—have been extensively studied for their rheological behavior, polymerization characteristics, and interfacial adhesion to dentin. The recent reformulation of AH Plus has stimulated renewed interest in its biomechanical properties, including solubility dynamics [8], adhesion to dentin [9], sealing capacity [10], antimicrobial efficacy [11], cytocompatibility [12], and clinical performance metrics [13]. The original AH Plus packaging—conical tubes—was problematic because it allowed the organic and inorganic components of the sealer to separate. This segregation led to unpredictable changes in critical properties like the setting time, flow, and radiopacity, as demonstrated by a previous study [14]. Dentsply/DeTrey addressed this by introducing the AH Plus Jet, which uses cylindrical syringes and mixing tips to ensure homogenous component mixing and simplify clinical use.

A frequent but obsolete method used for endodontic filling is represented by the single-cone obturation technique, considering the increased adoption of nickel–titanium (NiTi) rotary instrumentation and custom-milled tapered obturation gutta-percha cones [15]. A slightly newer method is the lateral compaction (LC) that ensures a better distribution of the sealer regarding the gutta-percha cones and the lateral unevenness of the radicular channels [16,17]. However, the sealing success strongly depends on the practitioner’s skill to ensure a proper fit of the cone within the channel.

Thermo plasticized gutta-percha is utilized in conjunction with warm lateral and vertical condensation, carrier-based systems, injection techniques, and thermo mechanical compaction methods [18]. It ensures proper expansion of the gutta-percha cone after its insertion, determining the sealer layer to fit the narrow space between the cone and the radicular channel irregular walls. That keeps the sealing layer slim and constant ensuring a better obturation [19,20]. Root curvature in the mesial and apical region represents a problem for the gutta-percha cones fitting, which must be compensated by thicker sealer layer but the WVC method ensure a proper obturation. Literature shows that the use of reciprocating instruments for filling removal and re-preparation was efficient in curved mesial canals of mandible molars [21].

The assessment of interfacial integrity, encompassing the evaluation of the gap morphology, void content, and material–dentin cohesion, has become a critical determinant of obturation quality. Modern analytical modalities, including radioisotope tracing, dye leakage, fluid filtration, bacterial penetration, light and electron microscopy, tissue-clearing techniques, and high-resolution imaging methods such as micro-computed tomography (micro-CT) and spiral CT, have significantly enhanced the capacity to quantify obturation performance [22,23,24]. Radiography and CT investigations can be effectuated in vivo for evaluating the patient state after the endodontic treatment. Usually, radiographic inspection is effectuated by the therapist just after the endodontic treatment is finished being enough to ensure the patient health. CT is more sophisticated technique, and it is not normally recommended for assisting the endodontic treatment and could be of great help in the scientific research, being able to collect reliable morphologic information and images at different sites of the obturation such as coronal, median, and apical sites without sectioning requirements. On the other side, scanning electron microscopy requires slices sectioning, but it allows a high precision measurement of the morphological details [25,26]. Literature data clearly reveal the benefit of the energy dispersive X-ray spectroscopy (EDX) associated with SEM investigations for revealing the mineral elements distribution in various samples, like environmental soil dispersions [27], but also on the dentistry samples, such as enamel and dentine interactions with the endodontic treatments [25,26].

The present investigation aims at an advanced microstructural investigation of the root canal endodontic sealing effectuated using three different methods, single cone (SC), lateral compaction (LC), and warm vertical condensation (WVC), and the same sealer, using X-rays, optical m, SEM, and the specific element distribution, which are evidenced based on EDX maps and spectra.

The null hypothesis was that there is no significant difference in the sealing ability or microstructural characteristics (including interfacial integrity, elemental composition, and sealer layer thickness and distribution) of the resin-based endodontic sealer AH Plus Jet when used with the three different root canal obturation techniques.

## 2. Materials and Methods

### 2.1. Sample Size Calculation

Based on the research of Akhtar et al. [16], the sample size calculation was performed a priori using G*Power 3.1.9.6 (Universität Kiel, Kiel, Germany), if a standardized effect size of 0.7276 should be detected via repeated measures ANOVA at 95% power and with a two-tailed probability of an alpha type error of 0.05. Finally, a sample of 20 teeth per group was selected, with a total of 60 teeth included in the study.

### 2.2. Samples Preparation

The study was conducted according to the guidelines of the Declaration of Helsinki and approved by the Ethics Committee of the “Iuliu Hatieganu” University of Medicine and Pharmacy, Cluj-Napoca, Approval number 10; Approval Date: 27 January 2025.

Sixty single-root teeth were included in the present study. The teeth were extracted for orthodontic or periodontal reasons 4 weeks before starting this experiment. The teeth that had internal root resorption, calcifications, or previous endodontic treatments or teeth that had been identified with more than one canal per root were removed from the study. For the batch uniformity, digital x-ray was performed with two different angles.

The teeth were coronary sectioned using a low-speed diamond disc, to obtain a 16 mm working length, identical in the whole studied group. The working length was determined visually by introducing a #10 K-file instrument (Dentsply, Stonehouse, Gloucestershire, UK) into the root canal until the tip was visible at the apex and then withdrawing the instrument for 0.5 mm.

Preparation steps:

a. Mechanical-antiseptic preparation of the root canals. The teeth were prepared at working length using the ProTaper Gold^®^ system (Dentsply, Stonehouse, Gloucestershire, UK) and X-Smart Plus Endodontic Endo Motor^®^ (Dentsply, Stonehouse, Gloucestershire, UK) in continuous rotation until the F2 instrument was at a speed of 250 rpm (revolutions per minute), according to the manufacturer’s instructions.

The mechanical and antiseptic treatment involved the use of chelating gel MM EDTA^®^ (Micro-Mega, Besancon, France) on each of the endodontic preparation instrument and continuous irrigation with a sodium hypochlorite (NaOCl) solution with a concentration of 2.5% (5 mL per sample), using a syringe gauge and side vented irrigation needle up to 3 mm of the working length. At the end of the preparation, the root canal was irrigated with 1 mL ethylenediaminetetraacetic acid solution (EDTA) at a 17% concentration and maintained for 3 min to remove the smear layer. Finally, the samples were irrigated with NaOCl 2.5% at 5 mL/canal under manual dynamic agitation. The canals were dried using paper points adjusted at the working length.

b. The root canal obturation. The sample of 60 teeth was randomly divided into three groups (*n* = 20). Each tooth of these groups was endodontically obturated using an epoxy resin-based sealer, the AH Plus Jet (Dentsply De Trey GmbH, Konstanz, Germany), and 3 filling techniques: single cone (SC), warm vertical condensation (WVC), and lateral compaction (LC).

All the endodontic procedures were performed by the same experienced operator with more than 15 years of clinical practice and were performed as follows:

A. Single cone technique: An ISO 40 standardized gutta-percha, 28 mm, taper 2% master point (VDW GmbH, Munich, Germany) was inserted into the canal, after its coating with the sealer. The cone was previously adapted (after confirming tug-back) into the canal. Then, using a heated instrument, the gutta-percha point was shorted.
B. Warm vertical condensation technique: Gutta-percha cones size 35, 0.05 taper (VDW GmbH, Munich, Germany) were used. The gutta-percha cone covered by the endodontic sealer was placed in the root canal. The thermoplastic plugger from the System B (Sybrondental, Orange, CA, USA), setting of 200 °C, using a Fine Plugger at 3.5 mm from working length, was used for plasticization, cutting, and compaction of the gutta-percha within the apical root canal up to 3 mm of the working length. After this, the thermal injector of the system was used to inject the warmed gutta-percha that was compacted.
C. Lateral compaction: Gutta-percha cones, size 35, 0.02 taper (VDW GmbH, Munich, Germany), coated with the endodontic sealer, were placed in the root canal. The finger spreader B (Dentsply Maillefer, Ballaigues, Switzerland) was used for inserting the auxiliary XF cones (VDW GmbH, Munich, Germany) into the apical third. Afterwards, the finger spreader C (Dentsply Maillefer, Ballaigues, Switzerland) was used for inserting the auxiliary FF gutta-percha cones (VDW GmbH, Munich, Germany) into the middle and coronal thirds.

To allow the complete setting of the sealer, the teeth were preserved in a saline solution with 100% humidity and a temperature of 37 °C for 7 days. After the complete setting of the sealer, the samples were placed in distilled water for 10 min, rinsed with absolute ethanol for 15 min, and then placed in an oven at a constant temperature of 37 °C for 24 h to dry them.

The samples, properly dried, were placed in resin blocks and left for 24 h to complete the setting reaction of the resin and then longitudinally sectioned using a microtome (Buehler-IsoMet 1000, Buehler Ltd., Lake Bluff, IL, USA) in slices of a 1 mm thickness.

### 2.3. Investigation Methods

Radiographic investigation was effectuated with the Planmeca ProX™ intraoral X-ray (Planmeca Oy Asentajankatu 6, Helsinki, Finland).

Optical microscopy was effectuated using the transmitted light method with a Zeiss microscope (Zeiss Company, Oberkochen, Germany) with a magnification of 75×. On each image, a qualitative analysis of voids was performed.

Scanning electron microscopy was effectuated with a Hitachi SU8230 (Hitachi Company, Tokyo, Japan) operated in high vacuum mode at an acceleration voltage of 30 kV. The morphological aspects were revealed by Secondary Electron Images (SEIs), while the elemental maps were overlying on the Backscattered Electron Images (BSEs) obtained with an X-Mas 1160 EDX detector (Oxford Instruments, Oxford, UK). The Energy Dispersive Spectra were taken on the specific areas to reveal their elemental composition. The teeth slices are electro-insulators and therefore are difficult to be observed via SEM at a high resolution; therefore, their surface was sputtered with a thin layer of Au for ensuring an optimal electrical conductivity, and the gold component was subtracted during the elemental investigation.

Systematic measurement of the sealing layer thickness was effectuated on the SEM images using specialized Image J software version 1.53k (National Institute of Health, Bethesda, Rockville, MD, USA). At least three independent measurements were effectuated, and the mean values were statistically analyzed by performing an Anova test followed by the Tukey post hoc, effectuated with Microcal Origin Lab version 2018b software (Microcal Company, Northampton, MA, USA).

## 3. Results

### 3.1. Periapical Radiographs

**Figure 1:**
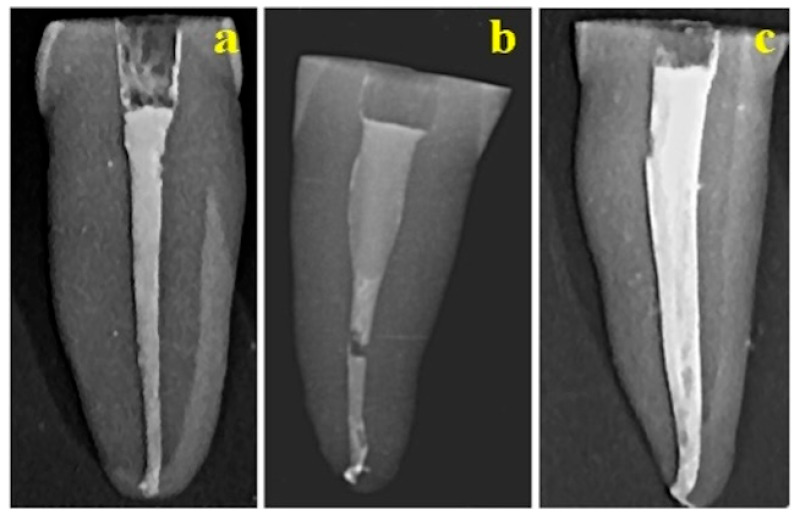
Radiographs of root canal fillings performed using the following methods: (**a**) SC technique, (**b**) WVC technique, and (**c**) LC technique.

Radiologic images of the endodontic fillings are displayed in Figure 1. In the single-cone (SC) obturation technique, the gutta-percha cone exhibits intimate adaptation to the canal walls through a uniform and thin layer of endodontic cement, thereby ensuring a hermetic seal and optimal dentin adhesion (Figure 1a).

The warm vertical compaction (WVC) specimens demonstrated extensive gutta-percha penetration into canal irregularities and superior interfacial adhesion; radiographic evaluation confirmed a continuous obturation with only minor discrepancies, such as a localized discontinuity in the median third and apical constriction, both of which merit further structural analysis (Figure 1b). In contrast, samples obturated using the lateral condensation (LC) technique displayed satisfactory gutta-percha adaptation and adhesion to dentin, accompanied by minor surface abrasions and halo-like formations. Nonetheless, the presence of possible micro cracks or discontinuities within the sealer layer suggests a potential for microleakage, underscoring the need for an advanced microscopic assessment to elucidate the extent of these defects (Figure 1c).

### 3.2. Optical Microscopy

**Figure 2:**
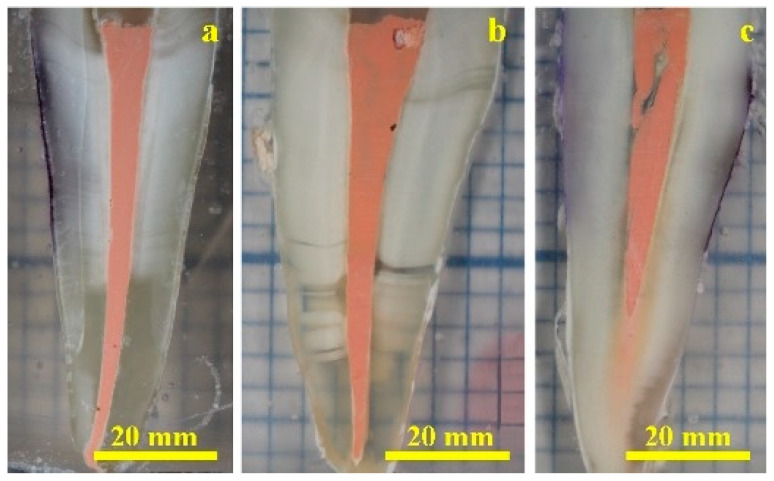
Optical microscopy images (magnification 75×) of root canal fillings performed using the following methods: (**a**) SC technique, (**b**) WVC technique, and (**c**) LC technique.

Sectioning of endodontically treated roots into thin transverse slices of approximately 1 mm thickness enabled a detailed observation under light microscopy, facilitating comprehensive visualization of the overall morphological and interfacial characteristics of the obturation complex (Figure 2). In specimens obturated using the single-cone (SC) technique, discrete microscopic discontinuities were occasionally observed along the gutta-percha–sealer interface (Figure 2a). Despite these localized gaps, the gutta-percha cone exhibited robust adhesion to the surrounding dentinal walls, mediated by a continuous cement layer of less than 0.5 mm in thickness. The apical third demonstrate particularly effective sealing, where the sealer uniformly conformed to the intricate topography of the dentinal surface, ensuring comprehensive coverage of all major microscopic features.

As illustrated in Figure 2b, sections obtained from the warm vertical compaction (WVC) group revealed an exceptional degree of homogeneity in the middle and apical thirds, characterized by intimate fusion between the gutta-percha mass and the dentinal substrate through a thin, macroscopically uniform cement layer extending over a length of approximately 68–69.5 mm. The apical region exhibited a densely compacted and conically shaped sealer plug, distinguished by its fine microstructural continuity and excellent interfacial adaptation, indicative of an effective apical seal.

In specimens obturated using the lateral compaction (LC) technique, the primary gutta-percha cone was observed to penetrate the prepared canal to a depth of approximately 53 mm, notably shorter than that achieved with the previously described obturation methods (Figure 2c). The remaining apical portion was predominantly occupied by a dense and continuous plug of the endodontic sealer, which contributed to satisfactory overall sealing performance. However, the cement layer enveloping the gutta-percha cone exhibited variable thickness, reaching values exceeding 1.5 mm in certain regions, suggestive of uneven material distribution during compaction.

Microscopic examination of the coronal third revealed a pronounced longitudinal incision within the gutta-percha mass, originating near the upper boundary of the median zone and extending obliquely toward the coronal surface at an angle of approximately 74° over 15 mm. This feature represents a significant microstructural discontinuity, which could potentially compromise the integrity of the obturation seal if the adjacent sealer layer fails to completely occlude the resulting gap within the root canal space.

### 3.3. Scanning Electron Microscopy and Elemental Analysis

#### 3.3.1. Single Cone Technique

**Figure 3:**
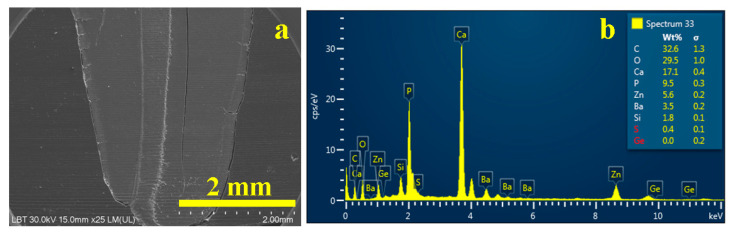
SEM image of the overall root canal filling performed using the MC method: (**a**) secondary electron (SE) image and (**b**) EDX spectrum with elemental composition.

Figure 3a indicates that the gutta-percha cone exhibited a smooth and homogeneous surface texture, firmly cemented to the adjacent dentinal substrate, indicative of excellent interfacial adaptation and a hermetic seal.

The corresponding energy-dispersive X-ray (EDX) spectrum (Figure 3b) revealed the elemental composition of the analyzed region, predominantly characterized by carbon (C) and oxygen (O) peaks, reflecting the high organic content inherent to the gutta-percha matrix and, to a lesser extent, the collagenous framework of dentin. The calcium-to-phosphorus (Ca/P) molar ratio was determined to be approximately 1.66, consistent with the stoichiometry of hydroxyapatite. The presence of barium sulfate (BaSO_4_) was confirmed, exhibiting a 1:1 stoichiometric ratio between barium (Ba) and sulfur (S), indicative of its role as a radiopacifying agent within the endodontic filling material [7,28].

**Figure 4:**
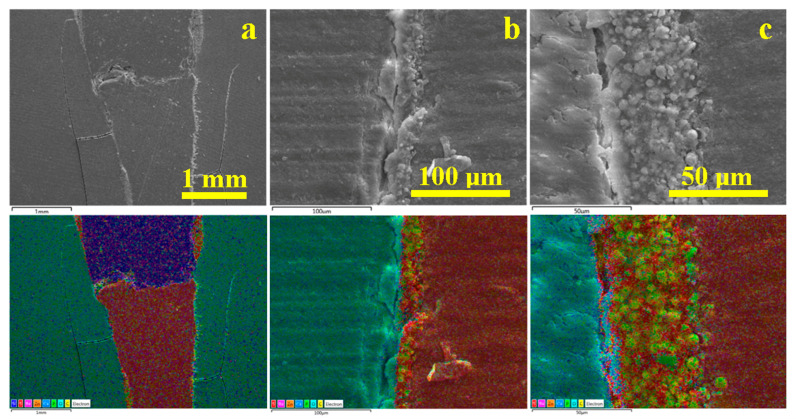
SEM images of backscattered electrons with the distribution map of the constituent elements of the main areas of the filling performed using the SC method: (**a**) coronal, (**b**) median, and (**c**) apical (green-turquoise areas—dentin walls, “blue cap with pink spots”—endodontic filling material, the gutta-percha cone appears burgundy red, yellowish line between the dentin and gutta-percha corresponds to the sealing cement).

Moreover, the detection of silicon (Si) was consistent with the sealer’s filler composition, as further corroborated by the elemental mapping shown in Figure 4. In this map, the distribution of the filling material is distinctly visualized, with regions of silica enrichment represented as dark blue matrices interspersed with pink particulate inclusions, delineating the microstructural heterogeneity of the sealer interface.

The middle area of the root canal treatment, Figure 4b, shows a compact and dense microstructure in both the dentin and gutta-percha areas. The sealer area has a local thickness of about 50 µm and highlights a granular material based on organic material with hydroxyapatite and traces of sulfur from the organic resin molecules. The hybrid layer is better developed in the apical region, as shown in Figure 4c. The light blue area in the elemental map indicates a relative thickness of the hybrid layer of 10–15 µm and shows its interpenetration with the dentinal tubules that have been sealed by the endodontic cement.

**Figure 5:**
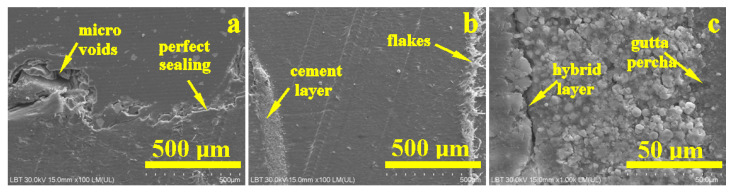
SEM–SE microstructural details for the area: (**a**) coronary, (**b**) median, and (**c**) apical.

The potential microscopic voids detected via optical microscopy on the interface between the gutta-percha and the endodontic filling material are clearly highlighted in Figure 5a. Some of the filler particles are highly visible, having a polyhedral appearance and dimensions of approximately 20–50 µm. Their shape and dimensions indicate that they are barium glass particles. On the left side of the junction (arrow), there are some abrasions on the upper part of the gutta-percha cone that interact unfavorably with the filler particles in the filling material. Properly, the filler particles cannot mold to the irregularities of the gutta-percha, leading to the formation of voids detected via optical microscopy. These cavities are elongated and dendritically branched over a length of 500 µm from the junction and would constitute ideal pathways for liquid penetration if the marginal seal of the gutta-percha cone were not watertight.

In the middle area, Figure 5b, the average thickness of the cement layer is 30–50 µm.

The thickness of the sealer layer increases slightly in the apical area, reaching almost 70–80 µm (Figure 5c). The microstructural detail on the left side of the image (arrow) shows how the hydroxyapatite content fuses with the demineralized collagen fibers on the dentin surface and forms the hybrid layer. On the right side, the well-laminated filler particles of the polymeric matrix perfectly coat the gutta-percha surface. The middle area of the image (arrow) clearly shows the microstructure of the endodontic cement with microstructural filler particles ranging in size from 3 to 15 µm, as well as much finer particles (most likely nanoparticles) of hydroxyapatite. All these filler particles are uniformly distributed in relation to each other and well embedded in the polymer matrix, which laminates them perfectly, generating a compact and sealed structure.

#### 3.3.2. Warm Vertical Condensation Technique

**Figure 6:**
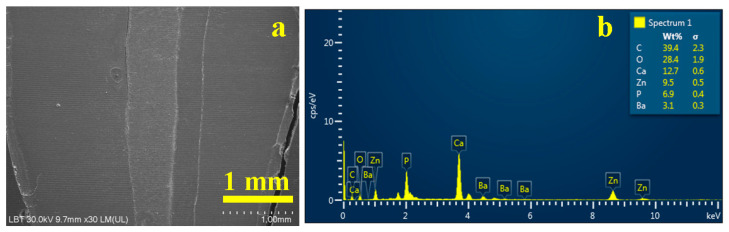
SEM image of the overall root canal filling performed using the WVC method: (**a**) secondary electron (SE) image and (**b**) EDX spectrum with elemental composition.

The root canal treatment using the WVC method presents an adequate overall appearance (Figure 6a) confirming the observations made under optical microscopy.

The EDX spectrum in Figure 6b indicates that C and O are dominant, partly due to the organic material of gutta-percha and the cement used, closely followed by Ca and P, which are key indicators of the presence of hydroxyapatite in dentin. The amounts of Zn and Ba detected correspond to the gutta-percha, being related to barium sulfate and zinc oxide. Sulfur, which appears in relatively small quantities compared to the other elements, is below the detection limit of the WVC method.

**Figure 7:**
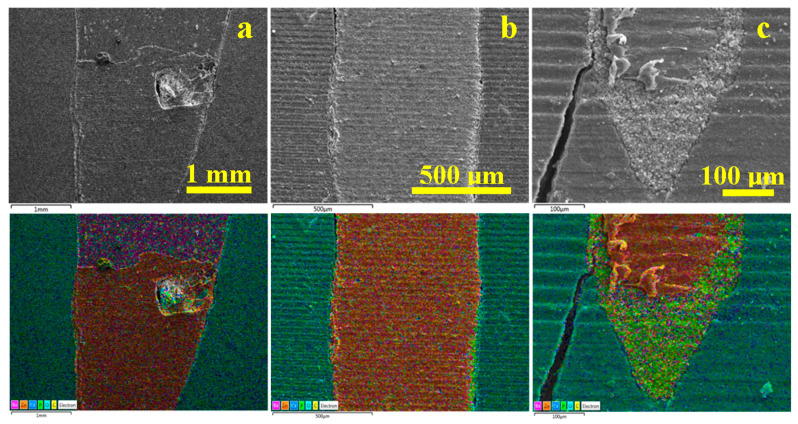
SEM images of backscattered electrons with the distribution map of the constituent elements of the main areas of the filling performed using the WVC method: (**a**) coronal, (**b**) median, and (**c**) apical.

For WVC samples (Figure 7) in the coronary area a very well-bonded junction between the gutta-percha cone and the endodontic filling material is observed, in which the filler particles interlock optimally with the gutta-percha without forming voids (Figure 7a).

The middle zone of the WVC samples (Figure 7b) indicates a monolithic unit of the sealer layer, which has a thickness of predominantly 35 µm, and the elemental map highlights the clear formation of the hybrid layer between the cement and dentin.

The apical area, Figure 7c, shows a sample artifact induced by the cracking of the tooth slice during sectioning; therefore, it must be disregarded when interpreting the microstructural aspects. The same cutting method also causes the three pieces of gutta-percha observed on the left side of the apical seal. Beyond these aspects, a strong bond is observed between the cement layer and the gutta-percha, as well as between the cement layer and the dentin. The hybrid layer is highlighted by the thin light blue line between the cement and the dentin. The very evident pistachio green hue of the cement is conferred by the predominance of carbon in the polymer matrix and phosphorus in the hydroxyapatite nano-filler.

**Figure 8:**
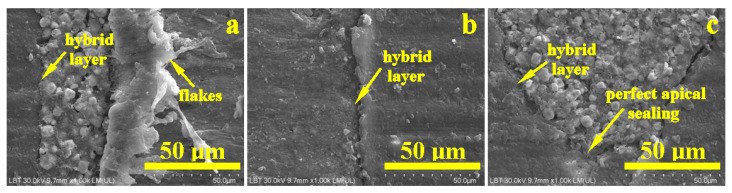
SEM–SE microstructural details for the area: (**a**) coronary, (**b**) median, and (**c**) apical.

Figure 8 reveals the microstructural details for each area of interest in the WVC root canal treatment. Thus, the coronal area shows a well-developed hybrid layer between the dentin and the endodontic sealer, with synergy between its Ca and P content and the demineralized collagen endings of the dentin surface. The thickness of this layer is approximately 10 µm, Figure 8a. The adhesion of the cement to the gutta-percha is very good, ensuring effective microstructural cohesion against fluid infiltration. The gutta-percha flakes, oriented to the right in Figure 8a, are due to mechanical action during cutting and do not affect the cohesion of the material. The thickness of the cement layer is around 35 µm.

The median area has a compact microstructure in which the layers involved are very well attached to each other, forming a monolithic whole, Figure 8b. The hybrid layer occurs at the junction between the cement and dentin and has a thickness of 10–15 µm, appearing as a longitudinal depression along the dentin wall with lateral interlocking to the right towards the dentin tubules that have been properly sealed.

Figure 8c highlights the bottom of the apical seal and shows only the conical cement plug that perfectly fills the canal termination. The 3–15 µm microstructural filler particles are very well laminated and embedded in the polymer matrix. On the other hand, the hybrid layer between the cement and dentin is very visible. Sealing the root canal termination is very important because it helps reduce the risk of microleakage.

#### 3.3.3. Lateral Condensation Technique

**Figure 9:**
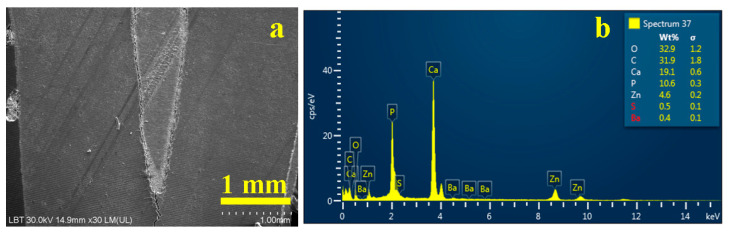
SEM image of the overall view of the root canal filling performed using the CVC method: (**a**) secondary electron (SE) image and (**b**) EDS spectrum with elemental composition.

The samples treated with LC showed some microstructural irregularities in the coronal part of the gutta-percha cone (Figure 9a).

The elemental composition of the sample is highlighted by the EDX spectrum in Figure 9b. In this case, C and O are also dominant, while Ca and P correspond to hydroxyapatite. This time, the elements Ba, S, and Zn are present, corresponding to the barium sulfate and zinc oxide introduced into the gutta-percha. The S and Ba content is very close to 0.5 and 0.4%, respectively, which corresponds to the stoichiometric ratio of barium sulfate. The fact that the EDX does not include the endodontic filling material above the gutta-percha cone in the investigated area deprives the EDX spectrum of additional barium content, as observed in the case of SC and WVC.

**Figure 10:**
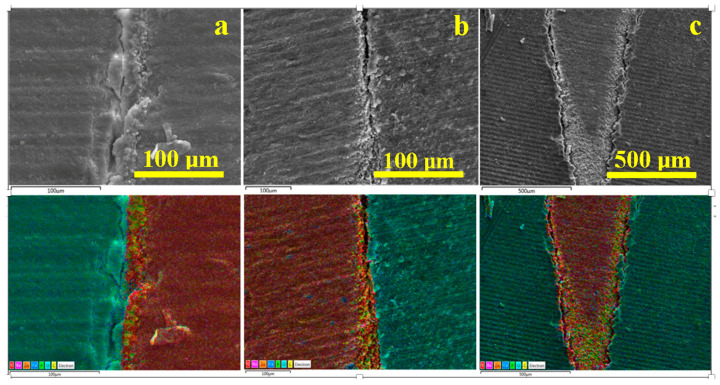
SEM images of backscattered electrons with the distribution map of the constituent elements of the main areas of the filling performed using the LC method: (**a**) coronal, (**b**) medial, and (**c**) apical.

The adhesion interface in the coronal area shows a 50 µm thick endodontic cement layer that adheres to the dentin surface through a hybrid layer of about 10 µm that appears light blue in the emission map corresponding to Figure 10a. The emission map shows a red interference over the yellow-green specific to orthodontic cement, indicating the presence of sulfur bonds in its mass, which facilitates better adhesion with gutta-percha.

In the middle section, the situation is similar, with the thickness of the endodontic sealer layer increasing progressively (Figure 10b). In the apical area, the cement layer forms a sealing plug under the tip of the gutta-percha cone.

**Figure 11:**
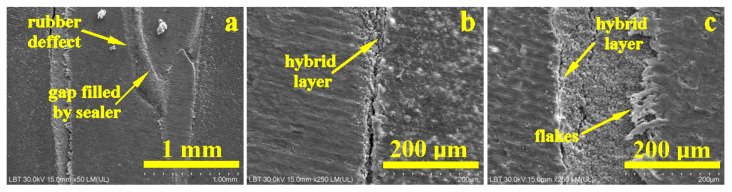
SEM–SE microstructural details for the area: (**a**) coronary, (**b**) median, and (**c**) apical.

Figure 11a highlights the microstructural details of the defect in the coronal part of the gutta-percha cone. It is very clear that its rubber mass was most likely torn accidentally by the endodontic instrument used to insert the cone into the canal. This tear has an upward trajectory, but fortunately, the endodontic sealer penetrated well into the cavity formed, perfectly sealing the entrance to the torn area, which contributes to the success of the filling’s tightness. Figure 11b,c show the increase in thickness of the endodontic sealer layer, as well as its perfect adhesion to the dentin wall through the hybrid layer and to the gutta-percha cone through excellent molding of the filler particles perfectly laminated by the polymer matrix.

**Figure 12:**
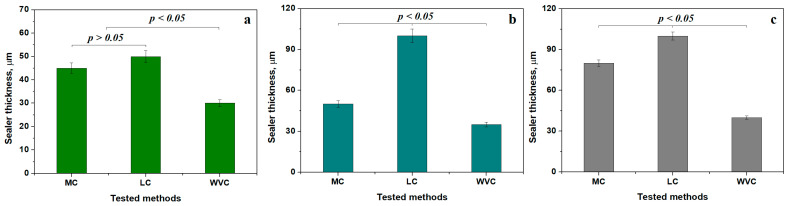
Variation of the mean thickness of the sealing layer for the targeted areas: (**a**) coronal, (**b**) median, and (**c**) apical.

The sealer layer thickness in each targeted areas (Figure 12) was also evaluated.

SC and LC ensure a thickness of about 45–50 µm in the coronal area forming a relevant statistical group (*p* < 0.05), which is in total discordance with the second statistical group consisting of the WVC method, which ensures a thin sealing layer of 35 µm, Figure 12a.

The canal irregularities increases along with the median area and its relative curvature inducing relative bigger distances between the dentin walls and the gutta-percha cones, which finally become full of sealer, a fact observed by the increased values observed for SC and LC, which do not form a statistical relevant group because the LC method seems to require a thickness of about 100 µm in the median side compared to SC, having only 50 µm, Figure 12b. The best results are also obtained using the WVC method, which keeps ensuring the sealer thickness at 35 µm. The statistical analysis reveals significant differences between all tested methods (*p* < 0.05).

The apical area is very important for sealing the bottom of the radicular canal and is often subjected to the formation of a clogged cluster of the sealer, increasing the local width of the layer, Figure 12c. Thus, SC has a thickness of 80 µm, while LC keeps an increased value of about 100 µm generating statistical differences between them. The thickness slightly increases at 40 µm for the WVC method.

**Figure 13:**
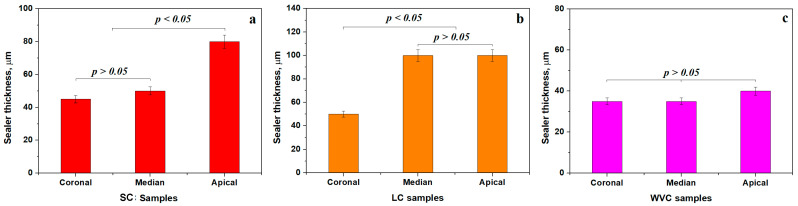
Variation in the mean thickness of the sealing layer for each tested method: (**a**) SC, (**b**) LC and (**c**) WVC.

The internal comparison for each method reveals that the coronal and median areas within SC form a relevant statistical group, having lower thickness, which increases only in the apical area, as observed in Figure 13a. It is good sealing behavior acting well against infiltrations for a long time. The LC method has a small thickness only in the coronal area forming a distinct relevant group, while the median and apical areas have a thicker sealer layer forming another relevant statistical group, proving the average results of this obturation. Finally, The WVC samples have about the same thickness in all areas forming a single relevant group proving the complete success of this method, Figure 13c.

## 4. Discussion

Although the quality of endodontic treatments continues to advance [29], the success rate for endodontically treated teeth has not yet reached complete reliability [30,31].

The current study aimed at investigating the AH Plus Jet (DENTSPLY Sirona, Charlotte, NC, USA) using three different endodontic obturation techniques (single cone (SC), lateral compaction (LC), and warm vertical condensation (WVC)). The AH Plus Jet is a resin-based sealer, considered the gold standard due to its physical properties, chemical properties, and clinical performance [32,33]. However, it cannot adhere to the gutta-percha and lacks bioactivity.

The null hypothesis that there is no significant difference in the sealing ability or microstructural characteristics (including interfacial integrity, elemental composition, and sealer layer thickness and distribution) of the resin-based endodontic sealer AH Plus Jet when used with the three different root canal obturation techniques was validated.

The EDX spectrum was used for material characterization, quality control, and failure analysis. Element composition analysis of AH samples showed elements such as C, O, Si, P, Zn, and Ca. The results from the current study were consistent with those of Abu Zeid et al., which showed that Ca, C, O, Si, Zr, and P were detected in samples of Cerafill and BC sealers [34].

In SC samples, the Ca and P ratio was 1.66. Ca and P are the most representative elements for hydroxyapatite (which is the main component of dentin). The Ca/P ratio obtained via the EDX measurement is very close to the stoichiometric ratio of 1.8, which shows a small excess of calcium that is most likely trapped in the organic material of the gutta-percha. Similar ratios were obtained for WVC and LC techniques.

Generally, in all analyzed samples, the stoichiometric ratio between Ba and S was 1:1. which indicates that only 0.4% of the detected Ba corresponds to the barium sulfate in the gutta-percha, and the remaining 3.1% corresponds to the Ba glass filler particles in the endodontic filling material placed above the gutta-percha cone. In LC samples, the EDX does not include the endodontic filling material above the gutta-percha cone in the investigated area, which deprives the EDX spectrum of additional barium content, as observed in the case of SC and WVC techniques.

The silica content also corresponds to the filling material, which is very evident in the elemental map. This indicates the homogeneous mixing of the siliceous material with the barium glass, ensuring a dense and compact composite material. The Zn content corresponds to the zinc oxide introduced into the gutta-percha as an antimicrobial agent at a percentage of 0.5%, and in quantities of up to 5%, it acts as a filler material that provides the mechanical consistency necessary for endodontic treatment.

A fusion of gutta-percha with the sealing cement was observed based on sulfur compounds. The endodontic cement used reacts with the collagen fiber endings in the partially demineralized dentin wall following the removal of the smear layer, facilitating its remineralization with hydroxyapatite and forming a hybrid layer that appears light blue in color due to its Ca and P content. The formation of this hybrid layer has the advantage of perfectly sealing the dentinal tubules, preventing the penetration of fluids into the canal space.

Complex microstructural analyses showed that each method used can ensure adequate sealing of the root canal, but success depends largely on the precision and accuracy with which the procedures were performed. The microstructural defects that were highlighted were related to minor handling accidents, especially of the gutta-percha cones, which suffered local microstructural alterations but were compensated for by the proactive effect of the endodontic sealer used. The local flaws of the cone insertion combined with the irregular and curved shape of the radicular channels were compensated by a thicker sealer layer which was not the optimal choice for a long-lasting endodontic restoration [35,36].

Overall, the MC method is relatively easy to be operated and has low costs, being affordable for a wide range of patients. The LC method is newer than MC and has significant control improvements and benefits, but the working procedures are more difficult requiring superior qualification of the therapist. WVC is the newest method, having relatively complicated procedures requiring novel devices and adequate materials, which increases their price, but the optimal results ensure that the endodontic sealing is long-lasting [37,38], while the other methods might be weakened in shorter time due to the progressive degradation of the resin-based sealer. It is a slow decaying process, which involves the progressive delamination of the filler particles and subsequent penetration of the liquid in the deeper layers down to the median or apical areas. The filler particle delamination process under liquid exposure stress is well described in the literature [39,40]. Thus, the smaller thickness of the sealing layer better resists the filler particle delamination and composite structure disintegration.

The main achievement of the present results is evidenced by the microstructural importance of the sealing layer thickness regarding the endodontic method used. The limitations of the current study primary refer to the ’in vitro’ nature of the study, which may not fully replicate the clinical conditions. Furthermore, the study focuses on a single resin-based sealer, and the results may not be generalizable to other types of sealers.

## 5. Conclusions

All tested methods ensure optimal sealing of the radicular channels through the development of a proactive hybrid layer between dentine and the sealer and the sealer gutta-percha cones. The WVC method ensures a uniform and thin sealing layer, achieving the best results, while the results obtained using the MC and LC methods seems to be influenced by the local geometry of radicular channels, requiring a thicker sealing layer, which further can be subjected to a shorter life time because of the risk of filler particle delamination and subsequent liquid infiltration from outer environment progressively through the coronal area down to the medial and apical zones.

## Data Availability

The original contributions presented in this study are included in the article. Further inquiries can be directed to the corresponding authors.
